# The Effect of Diurnal Fluctuation in Intraocular Pressure on the Evaluation of Risk Factors of Progression in Normal Tension Glaucoma

**DOI:** 10.1371/journal.pone.0164876

**Published:** 2016-10-24

**Authors:** Seung Hoon Kim, Eun Jung Lee, Jong Chul Han, Sae Woon Sohn, Taekkwan Rhee, Changwon Kee

**Affiliations:** Department of Ophthalmology, Samsung Medical Center, Sungkyunkwan University School of Medicine, Seoul, Korea; Oregon Health and Science University, UNITED STATES

## Abstract

**Purpose:**

To investigate whether diurnal fluctuation in intraocular pressure (IOP) can influence the result of the correlations between IOP-related factors and progression of normal tension glaucoma (NTG).

**Methods:**

Glaucoma progression was defined as visual field (VF) progression and changes in the optic disc and/or retinal nerve fiber layer (RNFL). Two different methods were used to evaluate the impact of the diurnal fluctuation in IOP. ‘Conventional method’ used in previous studies included all IOP measurements during the follow up time. ‘Time adjusted method’ was used to adjust diurnal fluctuation in IOP with the preferred time. Mean IOP, long term IOP fluctuation and the difference between the lowest and highest IOP were calculated using both methods. Cox regression analyses were performed to evaluate the association between IOP-related factors and NTG progression.

**Results:**

One hundred and forty eyes of 140 patients with NTG were included in this study. 41% (58 of 140 eyes) of eyes underwent NTG progression. Long term IOP variation calculated by conventional method was not a significant risk factor for NTG progression (hazard ratio[HR], 0.311; 95% confidence interval[CI], 0.056–1.717; *P* = 0.180). Long term IOP variation calculated by time adjusted method, however, was related to progression, with an HR of 5.260 (95% CI,1.191–23.232; *P* = 0.029).

**Conclusion:**

Although having the same IOP-related factors, if diurnal fluctuation is included, different results may be found on the relationship between IOP-related factors and NTG progression. Based on our results, diurnal fluctuation in IOP should be considered when IOP-related factors are studied in the future.

## Introduction

Intraocular pressure (IOP) is indispensable for the monitoring and treatment of glaucoma, and a higher IOP is a risk factor for the occurrence and progression of glaucoma.[1–5] Studies aiming to evaluate the relationship between IOP and glaucoma progression have used various concepts, such as mean IOP, peak IOP, short-term fluctuation and long-term IOP variation.

IOP is subject to diurnal fluctuation. The diurnal fluctuation described by Maslenikow in 1904 refers to the difference between the highest and lowest IOP in a single day. Several studies have reported that the diurnal fluctuation is higher in an eye with glaucoma than in a normal eye. Sihota *et al*. reported that the diurnal fluctuation in IOP in an eye with primary angle closure glaucoma (7.69 ± 3.03 mmHg) and an eye with primary open angle glaucoma (8.31 ± 2.58 mmHg) was larger than that in a normal eye (4.83 ± 2.46 mmHg).[6] The diurnal fluctuation in IOP in untreated normal-tension glaucoma (NTG) was significantly higher than that in a normal eye (3.9 ± 1.1 mmHg and 3.0 ± 2.0 mmHg, respectively).[7] Ishibashi *et al*. compared diurnal fluctuation in NTG patients before and after treatment with latanoprost. The diurnal fluctuation in IOP was 3.5 ± 0.9 mmHg before treatment and 2.7 ± 1.0 mmHg after treatment. De Vivero et al.[8] analyzed diurnal IOP fluctuation in 101 untreated NTG patients, of whom 77% had a peak IOP between 08:00 and 12:00 hours, inclusive. The peak IOP time is variable among individuals, as reported results include during daytime, early morning, and before noon.[9–11] Recent studies have taken into account postural variation and reported night-time IOP elevation.[12,13]

Knowing that diurnal fluctuation in IOP exist, most retrospective studies of the correlation between IOP-related factors with glaucoma progression have not considered the diurnal fluctuation in IOP. Glaucoma patients tend to be followed up for longer than those with other ophthalmological diseases. However, since the patients do not visit hospital at regular times, IOP is not measured at a constant time. Nevertheless, most studies of IOP-related factors judged glaucoma progression based on measurement of IOP once per day. In contrast, prospective studies measure IOP at particular times. Despite the difference in the time of IOP measurement between retrospective and prospective studies, no retrospective study has examined the correlation between IOP-related factors and the progression of NTG, taking into consideration the diurnal fluctuation in IOP.

This study aimed to examine the impact of the diurnal fluctuation in IOP on the correlations between IOP-related factors and progression of NTG. For this purpose, we examined the effect of IOP-related factors by correcting for the diurnal fluctuation in IOP on the progression of NTG. We also compared IOP-related factors determined by the conventional method used in previous studies of NTG progression with those identified using our time adjusted method.

## Methods

### Study population

Among 1663 patients diagnosed with NTG and treated solely with topical hypotensive agents at the Department of Ophthalmology at Samsung Medical Center from 2001 through to 2015, 140 patients who met the criteria were selected and reviewed retrospectively. This study followed a protocol that met the tenets of the Declaration of Helsinki and was approved by the Samsung Medical Center Institutional Review Board and Ethics Committee. We anonymized and de-identified the data before the analysis.

NTG was diagnosed if the patient had any of the following conditions: glaucomatous optic disc damage and corresponding visual field defects, normal open angle by gonioscopic examination, absence of IOP greater than 21 mmHg by Goldmann applanation tonometry without medication, and no underlying cause of optic disc damage other than glaucoma. Only patients who were treated with betaxolol were included, to exclude any confounding effect of treatment itself.

The criteria for inclusion in the study were as follows:

1. Aged between 30 and 80 years at the initial examination.
2. Follow-up period longer than 5 years.
3. Reliable visual field examinations (fixation loss <20%, false-positive and false-negative <15%) that were obtained at least five times.
4. Visual field examinations conducted more than twice per year.
5. Stereoscopic optic disc photographs more than once per year.
6. Best-corrected visual acuity higher than 10/20.
7. Treatment with betaxolol only without any previous use of other IOP-lowering medications.

If both eyes met the inclusion criteria, a study was conducted on the eye with the worse initial visual field examination result in the group without progression, and on the eye with greater progression in the group with progression.

The exclusion criteria were as follows:

1. Remarkable cataracts that may have influenced the visual acuity and visual field (cataracts were graded using The Lens Opacities Classification System III (LOCS III). Based on this grading, patients whose cataracts had progressed by more than C2, N2 and P2, were excluded from the analysis.
2. Refractive errors (spherical equivalent <-6.0 D or > +6.0 D, and astigmatism >2.5D).
3. Upon initial visual field examination, MD <-16 d B.
4. Other ophthalmic or neurologic diseases that might affect visual field examinations.
5. Overlap with other types of glaucoma, such as primary angle closure glaucoma, pseudoexfoliation glaucoma, and steroid-induced glaucoma.
6. Experience of intraocular surgery or laser treatment (with the exception of cataract surgery not accompanied by complications).

Initially, each patient underwent a comprehensive ophthalmic examination including a review of medical history, measurement of best-corrected visual acuity, slit lamp examinations, IOP measurement by Goldmann applanation tonometer, gonioscopic examinations, funduscopy, stereoscopic optic disc photographs, visual field examination and spectral domain optical coherence tomography.

### IOP measurement and definition of terminology

**Fig 1:**
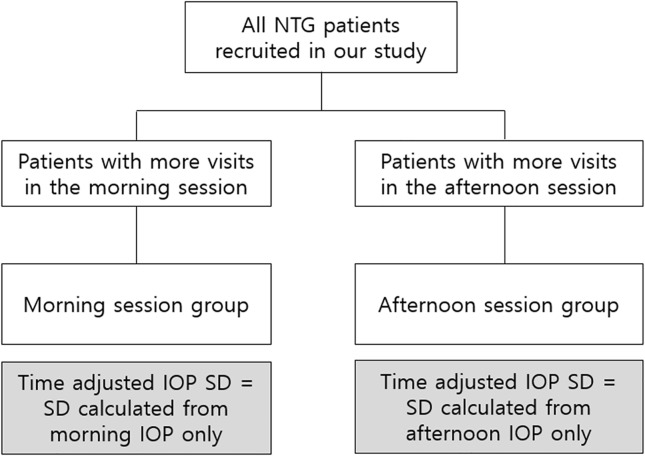
The selection method of patients based on more frequently visited session of time. Intraocular pressure (IOP) was divided into morning session (9 am-12 pm) and afternoon session (1 pm-5 pm) values. We identified a session with more frequent visit for each patient during the follow-up period, either morning or afternoon. The IOP and standard deviation of IOP measured during the selected session was defined as ‘time adjusted IOP’ and ‘time adjusted IOP SD’, respectively.

The subjects’ IOP was measured by a skilled examiner (CK) using Goldmann applanation tonometer during outpatient visits. In this study, we used two different methods to analyze IOP during the follow up period. The IOP determined by the ‘conventional method’ was the IOP measured during the follow up time. ‘Time adjusted method’ was used to adjust for diurnal fluctuation in IOP as follows. IOP was divided into morning session (9 am-12 pm) and afternoon session (1 pm-5 pm) values. We identified a session with more frequent visit for each patient during the follow-up period, either morning or afternoon. The IOP measured during the selected session was defined as ‘time adjusted IOP’. The selection method is demonstrated in Fig 1.

Basal IOP refers to the IOP measured before administration of topical anti-glaucoma medication.

Three IOP-related factors were evaluated in this study. First, the conventional mean IOP was the average IOP based on all IOP values measured during the follow-up period, while the time adjusted mean IOP was the average IOP of the selected sessions that were measured more frequently.

We defined long-term IOP differences as ‘long-term IOP variation’ and short-term diurnal IOP differences as ‘diurnal fluctuation.’ Conventional long-term IOP variation involved calculation of the SD of all measured IOPs, which was the method used in the study that compared the correlation between previous long-term IOP variation and glaucoma progression. In this study, we attempted to minimize the effect of diurnal fluctuation by using the time adjusted IOP SD. After selecting the session that were measured more frequently from the morning and the afternoon sessions, SD was calculated using IOP values of the more frequently measured session to define time adjusted long-term IOP variation. The difference between the lowest and highest IOP in the conventional method was defined as the difference between the highest and lowest IOP during the follow-up period, while the difference between the lowest and the highest IOP calculated using the time adjusted method was defined as the difference between the highest and lowest IOP after selecting the session that was more frequently measured from the morning or the afternoon session.

In the group without progression, analyses included all IOP measurements from the baseline visit to last follow-up. Only for patients with progression, we changed the treatment regimen; betaxolol was replaced with latanoprost or brimonidine or brimonidine/timolol. IOP recordings after change of medication were not included in the analysis to minimize the influence of confounding caused from altered treatments.

### Determination of progression

Glaucoma progression was judged based on functional and structural changes. First, to evaluate functional change, visual field examinations were performed using the Central 30–2 SITA standard strategy software of the Humphrey Field Analyzer (Model 750I; Humphrey Instruments Inc., San Leandro, CA). The results of the first two tests were not included to reduce the learning effect. Only a case in which there was ‘likely progression’ in the result of glaucoma progression analysis (GPA) was defined as functional progression.[14]

Structural changes in the optic disc and/or RNFL were identified by stereoscopic disc photography (Model TRC-50IX; Topcon Corp., Tokyo, Japan). The optic disc committee, which was composed of three ophthalmologists (CK, JCH, SHK), evaluated the changes in optic discs and RNFLs. The baseline and follow-up photographs were analyzed in a blinded fashion condition. Changes in RNFL defects, and general or local thinning of the neuroretinal rim were defined as structural changes, but disc hemorrhage was not. Results were derived through consultations when a consensus could not be reached.[15]

Only cases in which functional and structural changes were evident was included in the progression group. In the progression group, IOP values were collected from the records up to the time at which NTG worsened based on the GPA of visual field examinations. We applied a strict criterion to judge the presence of progression. When equivocal degrees of functional changes were observed, we followed the patients while repeating VF tests and finally confirmed the presence of progression when corresponding structural changes were identified. When structural changes were present without VF changes, we re-examined the VF tests until we could observe the corresponding VF changes, and confirm the progression.

### Data analysis

The primary purpose of the study was to establish whether diurnal fluctuation in IOP affects the relationship between IOP-related factors and NTG progression. For this purpose, using IOP-related factors with and without adjustments for diurnal fluctuation in IOP as explanatory variables, this study examined the effect of long-term IOP variation expressed as SD of IOP, in both conventional method and our time-adjusted method to minimize the effect of IOP fluctuation. In addition, we investigated whether sex, age, central corneal thickness (CCT), baseline visual field mean deviation (MD), pattern standard deviation (PSD) and visual field index (VFI) of the Humphrey field analyzer were predictive factors. For hazard ratios (HRs) of the association between predictive factors and NTG progression, univariate models lacking other corrected factors were performed, followed by multivariate Cox proportional hazards models to remove confounding factors.

For long-term IOP variation without adjustment for diurnal fluctuation (conventional long-term IOP variation, CLT), the subjects were divided into groups with fluctuations of >1.5 mmHg and <1.5 mmHg, and NTG progression was analyzed using the Kaplan-Meier analysis and the log-rank test to compare survival experiences (time-to-progression event).[16] In addition, to assess long-term IOP variations after adjustment for diurnal fluctuation (time adjusted long-term IOP variation, TALT), the subjects were divided into groups with variations of >1.5 mmHg and <1.5 mmHg, and a survival analysis was conducted.

Data analysis was conducted using the SPSS software version 23.0 (SPSS, Chicago, IL, USA).

## Results

One hundred and forty eyes of 140 patients with NTG were included in this study. Glaucoma progression was confirmed by visual field examinations, disc photo and/or RNFL change in 58 patients, while 82 patients did not exhibit progression. The demographics and clinical characteristics of the 140 eyes of 140 NTG patients included in this study are shown in Table 1. The mean age of those in the glaucoma progression group was 53.8±15.5 years, while that of those in the group without progression was 58.1±15.5 years. Baseline IOP was 15.3±2.5 mmHg in the group without progression, and 15.5±2.8 mmHg in that with progression. The MD value in the visual field examination at the time of diagnosis was -4.14±3.84 dB in the group without progression and -3.64±3.13 dB in that with progression, the difference was not significant. Moreover, no significant difference was found between the two groups in terms of PSD and VFI values. Distributions of sex, diabetes, hypertension, spherical equivalent and CCT are presented in Table 1. There were no significant differences in these baseline factors between the two groups.

**Table 1:** Clinical factors of NTG patients with and without progression.

	**Non-progression (***n* **= 82)**	**Progression (***n* **= 58)**	*P*
Age (y)	53.8 ± 14.3	58.1 ± 15.5	0.071^*^
Male, n (%)	43(51.8)	25(43.9)	0.400†
DM, n (%)	8 (9.6)	5 (8.8)	0.732†
HTN, n (%)	11 (13.3)	12 (21.1)	0.209†
Refraction (D)	-1.85 ± 2.5	-2.0 ± 2.5	0.750^*^
CCT (μm)	531.7 ± 34.5	521.6 ± 33.3	0.089^*^
Baseline IOP (mmHg)	15.3 ± 2.5	15.5 ± 2.8	0.699^*^
Baseline MD (dB)	-3.60 ± 3.13	-4.14 ± 3.83	0.375^*^
Baseline PSD (dB)	6.17 ± 4.26	6.46 ± 4.18	0.694^*^
Baseline VFI (%)	90.64 ± 9.34	88.22 ± 11.12	0.179^*^

DM, Diabetes Mellitus; HTN, hypertension; D, diopter; CCT, central corneal thickness; IOP, intraocular pressure; MD, mean deviation; PSD, pattern standard deviation; dB, decibel; VFI, visual field index.

*Student’s t-test.

† Chi-squared test.

**Fig 2:**
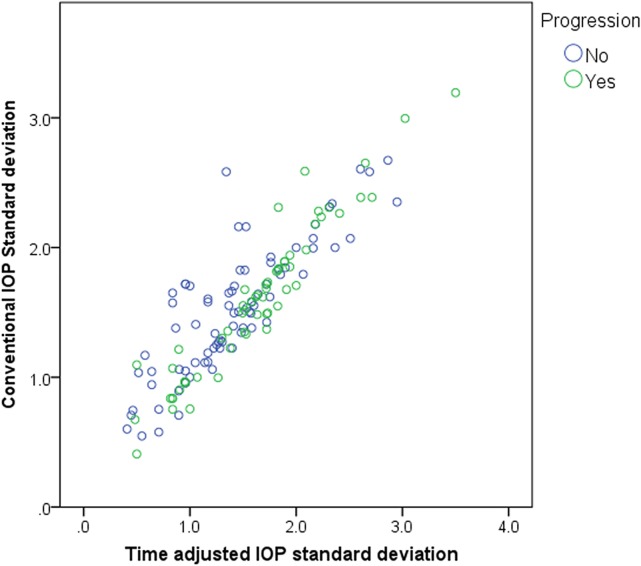
Relationship between conventional and time adjusted intraocular pressure (IOP) standard deviations (SDs). The two variables were significantly related to each other, but deviation from direct proportion is more common in non-progression group. The ratio between conventional IOP SD and time adjusted IOP SD was significantly smaller in progression group than non-progression group (*P* = 0.002).

Table 2 demonstrates the percentage of visits performed in the more frequently visited session, morning or afternoon session. The majority of patients visited in consistent session of time. Conventional IOP SD was 1.554±0.536, and time adjusted IOP SD was 1.503±0.602, which was significantly different (*P* = 0.028). The relationship between conventional and time adjusted IOP SD is shown in Fig 2. The ratio between conventional IOP SD and time adjusted IOP SD (conventional IOP SD/time adjusted IOP SD) was 1.005±0.201 for progression group, and 1.146±0.314 for non-progression group, and the value was significantly larger in the non-progression group (*P* = 0.002).

**Table 2:** Percentage of used values of intraocular pressure measurements according to more frequently visited session, morning or afternoon type.

	Morning type (n = 82)	Afternoon type (n = 58)	Total (n = 140)
Percentage of used visits (%)	82.0 ± 16.0	78.3 ± 15.8	80.5 ± 15.9
Mean IOP from used values	14.8 ± 2.28	14.5 ± 2.22	14.7 ± 2.3
SD IOP from used values	1.50 ± 0.58	1.50 ± 0.64	1.50 ± 0.60
Mean IOP from unused values	15.2 ± 2.4	14.7 ± 2.1	15.0 ± 2.3
SD IOP from unused values	1.32 ± 0.92	1.21 ± 0.80	1.27 ± 0.87

IOP, intraocular pressure; SD, standard deviation

Table 3 shows univariate HRs of risk factors for progression of NTG. Disc hemorrhage was a significant predictive factor for NTG progression in a univariate analysis (HR, 2.863; 95% confidence interval [CI], 1.676–4.893; *P* < 0.001). Of the IOP-related factors, only the TALT was a significant predictive risk factor (HR, 1.629; 95% confidence interval [CI], 1.063–2.496; *P* = 0.025). In contrast, conventional mean IOP and CLT were not significantly predictive of NTG progression.

**Table 3:** Cox proportional hazards model of progression (univariate analysis).

	**Hazard Ratio**	**95% CI**	*P*
Age (y)	1.002	0.984–1.020	0.805
Sex	0.675	0.399–1.141	0.142
Refraction (SE, D)	0.962	0.861–1.075	0.497
HTN, n (%)	0.908	0.660–1.250	0.555
DM, n (%)	1.159	0.730–1.838	0.532
Baseline MD (dB)	1.000	0.928–1.077	0.992
Baseline PSD (dB)	0.990	0.931–1.054	0.756
Baseline VFI	0.994	0.969–1.018	0.610
Disc hemorrhage	2.863	1.676–4.893	<0.001
Conventional Mean IOP	1.081	0.959–1.219	0.201
Time adjusted Mean IOP	1.073	0.955–1.205	0.237
Conventional SD of IOP	1.343	0.823–2.191	0.237
Time adjusted SD of IOP	1.629	1.063–2.496	0.025
Conventional difference between lowest and highest IOP	1.014	0.871–1.180	0.860
Time adjusted difference between lowest and highest IOP	1.105	0.963–1.269	0.153
CCT	0.996	0.989–1.004	0.362

D, diopter; HTN, hypertension; DM, Diabetes Mellitus; MD, mean deviation; PSD, pattern standard deviation; dB, decibel; VFI, visual field index; IOP, intraocular pressure; SD, standard deviation; CCT, central corneal thickness

Table 4 shows the result of multivariate analyses from which factors with a *P* > 0.2 were excluded to remove confounding factors. In this model, disc hemorrhage (HR, 0.325; 95% confidence interval [CI], 0.180–0.583; *P* < 0.001) and TALT (HR, 5.260; 95% confidence interval [CI],1.191–23.232; *P* = 0.029) were predictive of NTG progression.

**Table 4:** Cox proportional hazards model of progression (multivariate analysis).

	Hazard Ratio	95% CI	*P*
Age	1.009	0.987–1.032	0.418
Sex	0.643	0.344–1.204	0.168
Baseline IOP	0.928	0.786–1.095	0.376
Conventional mean IOP	1.184	0.941–1.489	0.150
Conventional IOP SD	0.311	0.056–1.717	0.180
Time adjusted IOP SD	5.260	1.191–23.232	0.029
Disc hemorrhage	3.082	1.174–5.541	<0.001
Baseline visual field index	0.985	0.959–1.013	0.285
Central corneal thickness	0.999	0.988–1.010	0.858

IOP, intraocular pressure; SD, standard deviation

**Fig 3:**
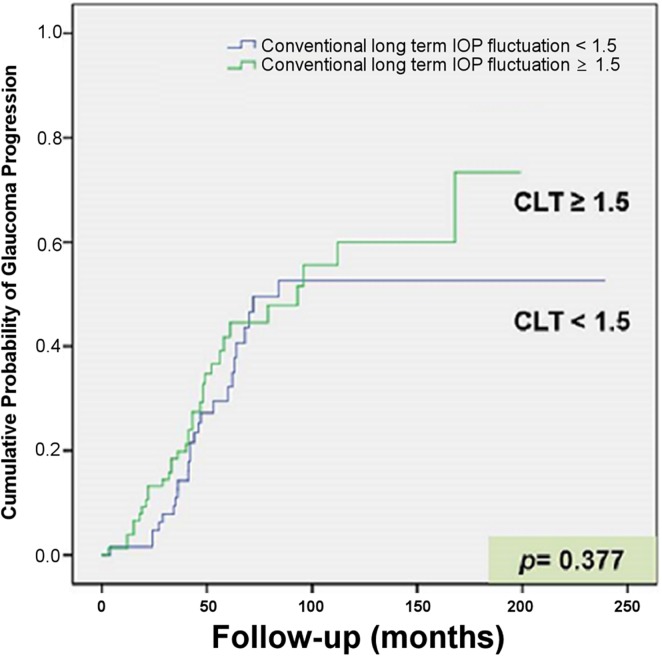
Kaplan-Meier curve showing the cumulative probability of glaucoma progression in patients with NTG. With conventional long-term IOP variation (CLT), no difference in the cumulative probability of glaucoma progression was detected between the groups with a standard deviation of IOP of >1.5 and <1.5 mmHg (*P* = 0.377, log-rank test).

**Fig 4:**
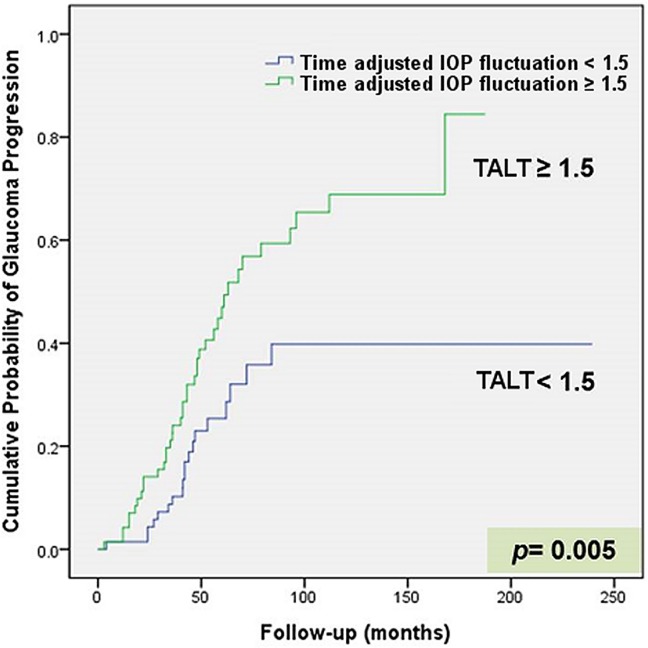
Kaplan-Meier curve showing the cumulative probability of glaucoma progression in patients with NTG. With time adjusted long-term IOP variation (TALT), the group with long-term IOP fluctuation of >1.5 mmHg had a higher cumulative probability of glaucoma progression than the group with standard deviation of IOP < 1.5 mmHg (*P* = 0.005, log-rank test).

A Kaplan-Meier curve of the cumulative probability of NTG progression is shown in Fig 3. When the survival rate was compared between the groups with a CLT of >1.5 and <1.5, there was no significant difference (*P* = 0.377, log-rank test). In contrast, the cumulative probability of NTG progression was higher in the group with a cumulative probability of TALT of >1.5 than in the group with a cumulative probability of TALT of <1.5, which was statistically significant by log-rank test (*P* = 0.005, Fig 4).

**Fig 5:**
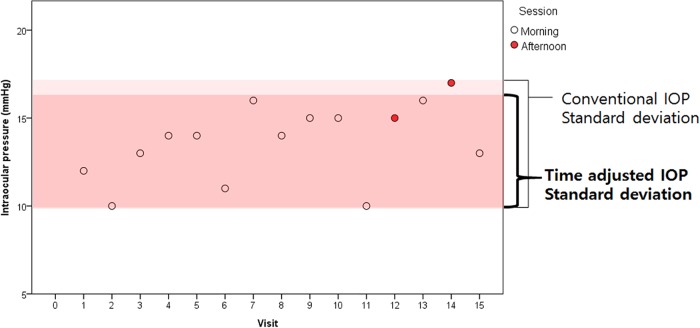
An example demonstrating the effect of diurnal fluctuation on the overall standard deviation value of intraocular pressures (IOPs) during follow-up in an individual patient. This patient was a 47-year-old male who was diagnosed as normal tension glaucoma for the both eyes. The graph demonstrates the IOPs of the right eye on his continuous follow-up. The distribution of this patient’s IOP in the whole follow-up period was 13.67 ± 2.16 mmHg. When the IOP distribution was calculated only for the morning session values the result was 13.87 ± 1.46 mmHg, which showed a lower standard deviation value. He showed no evidence of progression during the total follow-up period of 144 months.

Fig 5 illustrates an example of inadequately exaggerated SD value of IOP when IOPs from different time session are included in the calculation of SD.

## Discussion

This study was based on the hypothesis that diurnal variation, which is a well-proven characteristic of IOP, has an important effect on the correlations between NTG progression and IOP-related factors. If the effects of IOP-related factors on NTG progression after time dependent adjustments for diurnal fluctuation of IOP were different from those of conventional IOP-related factors, diurnal fluctuation will facilitate identification of correlations between IOP-related factors and NTG progression.

In our study, IOP SD (long-term IOP variation) was a significant factor in NTG progression, while IOP SD calculated using a conventional method was not. Therefore, IOP SD is influenced by the diurnal fluctuation of IOP, which should be considered when assessing the correlations between IOP-related factors and NTG progression.

The results of previous studies on the correlations between IOP SD and glaucoma progression are controversial.[2,17–22] Nouri–Mahdavi et al.[18] proposed that odds of visual field progression in glaucoma patients increase by 30% for each 1 mmHg increase in IOP SD in AGIS data; Lee et al.[19] also stated that visual field progression increases 4.2-fold for each 1 mmHg increase in IOP SD in patients with primary open-angle glaucoma (POAG) including NTG and ocular hypertension (OHT). In contrast, Bengtsson et al.[2] stated that correlations of IOP SD with glaucoma progression could not be determined using EMGT; and Medeiros et al.[21] reported results similar to those of EMGT. Fukuchi et al.[20] reported that rapid progression is related to high follow-up IOP in high-tension glaucoma, while long-term IOP variation is involved in NTG. According to Medeiros et al., the different correlations between IOP SD and glaucoma progression are attributable to use of different inclusion and exclusion criteria and depend on whether IOP after glaucoma progression is included.[21] This study confirmed that diurnal fluctuation in IOP also has a significant effect on identifying those correlations.

Moreover, treatment of glaucoma can also have effects on IOP SD. If glaucoma progression is identified, in general, medication is changed or added. Moreover, if there is no response to medical therapy, surgeries are performed to further lower IOP. IOP fluctuation in this case can result in changes in IOP SD. As the major purpose of this study was to analyze the effects of diurnal fluctuation in IOP, patients who used only betaxolol with no experience of using other IOP-lowering medications at the first examination or glaucoma filtering surgery were chosen as subjects. In other words, we applied strict inclusion criteria to minimize the influence of confounding factors.

Diurnal fluctuation in IOP is difficult to measure. Currently, the most accurate method of measuring diurnal fluctuation in IOP is to monitor 24-hour changes in IOPs of hospitalized patients.[23,24]

As mentioned above, although measuring the diurnal fluctuation in IOP is problematic, it is necessary for the following reasons. First, IOP measured once in the sitting position during a hospital visit cannot represent the diurnal fluctuations of IOP.[25] A study analyzed the diurnal fluctuation of IOP in 524 suspected NTG patients and found their mean diurnal fluctuation to be 4.4 ± 1.6 mmHg. Of the subjects, 57.9% showed diurnal fluctuation of less than 4 mmHg and 24.4% of more than 6 mmHg, suggesting that a large number of suspected NTG patients had a greater diurnal fluctuation than suspected.[26] By definition, NTG patients had baseline IOP lower than those of POAG patients; their IOP is less than 21 mmHg. Thus diurnal fluctuation should be considered because even a diurnal fluctuation of 4.4 mmHg can have a marked effect. Lastly, diurnal fluctuations typically peak in the morning and early afternoon, while trough in the afternoon and at night.[26–28] Thus IOP measured in the morning is likely close to the peak IOP while that measured in the afternoon is close to the trough IOP. Therefore, calculation of long term IOP variation that involved measurement of IOP both in the morning and afternoon should take into account the diurnal fluctuation when interpreting the IOP recordings.

In many cases, IOP measurement time is not considered in studies of IOP-related factors. However, as mentioned above, IOP measured only once in a sitting position during the clinic hour cannot represent the diurnal fluctuation of IOP. Moreover, identifying accurate correlations between IOP-related factors and glaucoma progression without considering diurnal fluctuation is problematic due to the relatively large diurnal fluctuation in NTG. According to preferred time, IOP was classified as time adjusted (diurnal fluctuation was adjusted in a time dependent manner) or conventional (diurnal fluctuation was not adjusted) in this study. Whether it was morning or afternoon does not affect the magnitude of standard deviation, which was our focus of analysis while it might possibly affect the mean value. Therefore, we considered our method would not add bias in the analysis. Based on this method, we investigated the correlations between IOP-related factors and glaucoma progression and obtained different results. Thus our study demonstrated the importance of considering diurnal fluctuation of IOP in evaluation of IOP-related factors for the first time.

This study was limited by the small number of subjects. Many NTG patients were being treated in the glaucoma clinic of our hospital, but only 140 were enrolled in this study based on the inclusion criteria. For example, subjects were required to have used only betaxolol and had undergone follow up of more than a certain period. A further limitation was that advanced glaucoma patients were not included because their MD value was less than -16 dB during their visual field examination. However, our study is meaningful as it involved a limited set of subjects and the possible influence of confounding factors was controlled for.

In addition, the method of correcting diurnal fluctuation was selected using only the session in which a greater number of IOP measurements were performed because diurnal fluctuation could not be accurately controlled due to the retrospective nature of the study. This method does not stand for the gold-standard adjustment of diurnal fluctuation. But in this study, it could demonstrate a difference in significance of IOP-related risk factors. Therefore, we speculate that our method of time adjustment might possibly be meaningful in assessing the effect of IOP variation in the clinical field.

In conclusion, the long term IOP variation adjusted for diurnal fluctuation was significantly related to glaucoma progression in NTG patients while the IOP variation by conventional calculation was not. On evaluation of IOP-related factors for glaucoma progression, diurnal fluctuation can lead to different results. Thus, diurnal fluctuation of IOP should be considered to more accurately evaluate the effect of various IOP-related risk factors for glaucoma progression, especially the long-term IOP variation. Further prospective studies designed with fixed time visits are warranted in the future.

## Supporting Information

S1 Fig Age distribution of the study population. (TIF)

S2 Fig Follow-up duration of the study population. (TIF)

S3 Fig Refractive error of the study population. (TIF)

S4 Fig Baseline intraocular pressure of the study population. (TIF)
