# Supplementary figures and images for: The Effect of Diurnal Fluctuation in Intraocular Pressure on the Evaluation of Risk Factors of Progression in Normal Tension Glaucoma
Source: PLoS One. 2016 Oct 24;11(10):e0164876. doi: 10.1371/journal.pone.0164876 (PMC5077094; doi:10.1371/journal.pone.0164876)

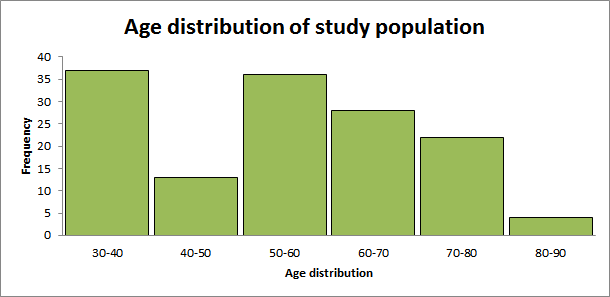

Supplement: S1 Fig — (TIF) [file pone.0164876.s001.tif]

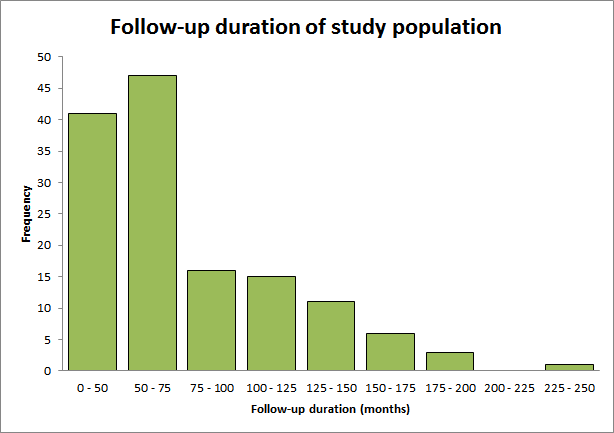

Supplement: S2 Fig — (TIF) [file pone.0164876.s002.tif]

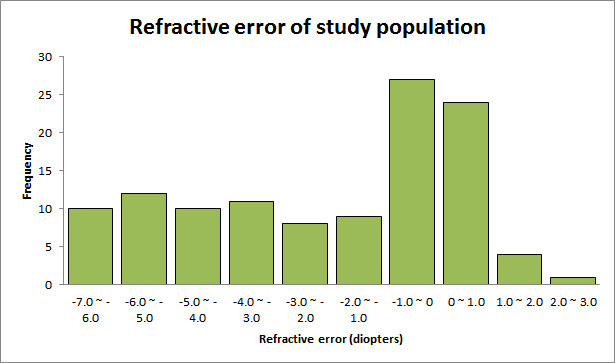

Supplement: S3 Fig — (TIF) [file pone.0164876.s003.tif]

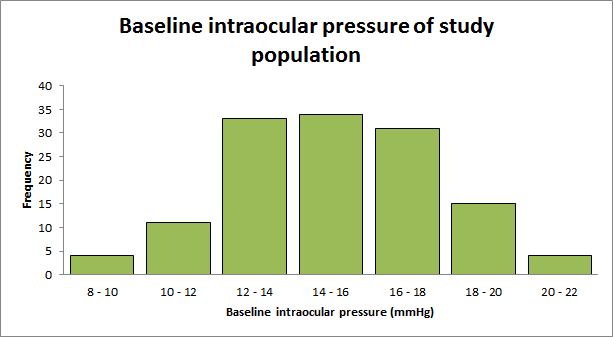

Supplement: S4 Fig — (TIF) [file pone.0164876.s004.tif]
